# Experimental data showing the thermal behaviour of a residential building in a hot and humid climate on three scenarios: An empty room with a closed door, an empty room with an open door, and a normal inhabited room

**DOI:** 10.1016/j.dib.2022.107906

**Published:** 2022-02-08

**Authors:** Thomas Janvier Matongo, Jean Gaston Tamba, Léopold Mba, Emmanuel Yamb

**Affiliations:** a Laboratory of Technologies and Applied Science, University Institute of Technology, University of Douala, Douala, Cameroon; b Laboratory of Mechanics and Adapted Materials LAMMA, Advanced Technical Teachers Training College, University of Douala, Douala, Cameroon; c Department of Electrical Engineering, Advanced Technical Teachers Training College, University of Douala, Douala, Cameroon; d Department of Civil Engineering, Advanced Technical Teachers Training College, University of Douala, Douala, Cameroon

**Keywords:** Data prediction, Causal links, Residential building, Thermal behaviour, Hot and humid climate

## Abstract

Data presented on this paper are from the collection on the experimental site during a 12 months period, corresponding to the two seasons of the year. The site is located in the city of Douala in Cameroon, in a hot and humid zone. The experimental premises are located above the ground floor of a storey-building. They are built of 20 cm thick hollow breezeblocks with interior and exterior cement plastering, and they consist of the following: an open uninhabited room; a closed uninhabited room; and an inhabited room. The data acquisition system was achieved by the thermo-hygrometers and the anemometer placed in the rooms in accordance with the ASHRAE 55 standard [1], on instruments for measuring thermal comfort parameters in buildings. Collected data consists of the following: temperature, relative humidity and air velocity outside the site; the temperature and relative humidity inside the different rooms. These parameters are collected with a one-hour time step. The matlab software is used to calculate the maximum, minimum, average and standard deviation for each measured parameter. several areas are used for data processing: the search for causal links between the climatic parameters of the site, and those of the indoor environments of the buildings; data prediction on a one year's history basis; the impact of each experimental scenario on the thermal behaviour of the room; the assessment of the heat transfer in the room; the evaluation of the potential for energy savings in the room.

## Specifications Table

**Table:** 

Subject	Physics
Specific subject area	Energy Building.
Type of data	csv data, figure, image
How data were acquired	Data were collected using the following instruments: - Thermo hygrometers, model EXTECH RHT10 - Anemometer, model WINDVISU
Data format	Raw data
Parameters for data collection	Data were collected in the climatic environments outside and inside the experimental premises located in a hot and humid climate. The premises consisted of three rooms: an uninhabited open room, an uninhabited closed room, and an inhabited room.
Description of data collection	Temperature and relative humidity data as well as wind speed were collected with an hourly time step, and over a 12 months period, corresponding to the two seasons of the year.
Data source location	Institution: University of Douala Country: Cameroon Latitude and longitude (and GPS coordinates, if possible) for collected samples/data: 4.0193785; 9.8006592
Data accessibility	With the article as a supplementary file.

## Value of the Data

• Data collected is useful for researching the causal links between climatic parameters of the site and those of the indoor environment of buildings [2,3].
• Engineers and researchers in the field of energy, environment and civil engineering can benefit from the data as it can be used to predict energy consumption in residential buildings.
• Data will be used to: develop models of thermal behaviour of the building in hot and humid climate; predict climatic parameters on the basis of 12 months of collected data; establish correlations between the collected parameters; simulate energy saving potential [4].

## 1 Data Description

Data collected is grouped into 12-month series simultaneously in the three experimental rooms.

‐ Room C1 is open to the outdoor environment and is uninhabited.
‐ Room C2 is closed and is uninhabited.
‐ Room C3 is inhabited.

Each month includes hourly values of nine parameters:

‐ The outdoor air temperature, Tout.
‐ The outdoor air relative humidity, RHout.
‐ Maximum wind speed, Vout.
‐ The indoor air temperature of room C1, TC1.
‐ The indoor air relative humidity of room C1, RHC1.
‐ The indoor air temperature of room C2, TC2.
‐ The indoor air relative humidity of room C2, RHC2.
‐ The indoor air temperature of room C3, TC3.
‐ The indoor air relative humidity of room C3, RHC3.

The hourly data for each parameter are presented in the following Table 1 corresponding to the months of January to December:

**Table 1:** Data for the month of January to December.

**Fig. 1:**
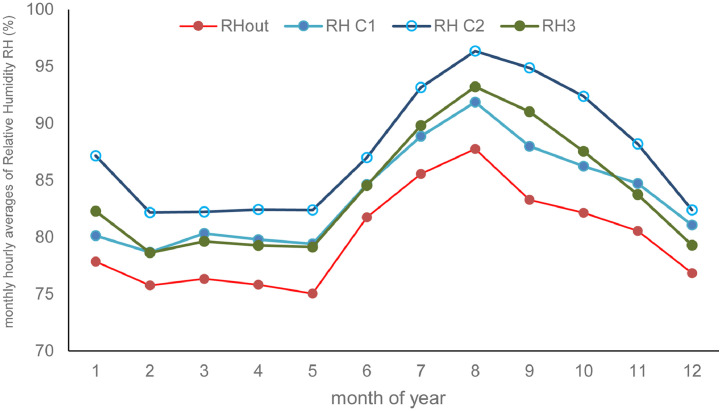
Monthly hourly average of relative humidity.

**Fig. 2:**
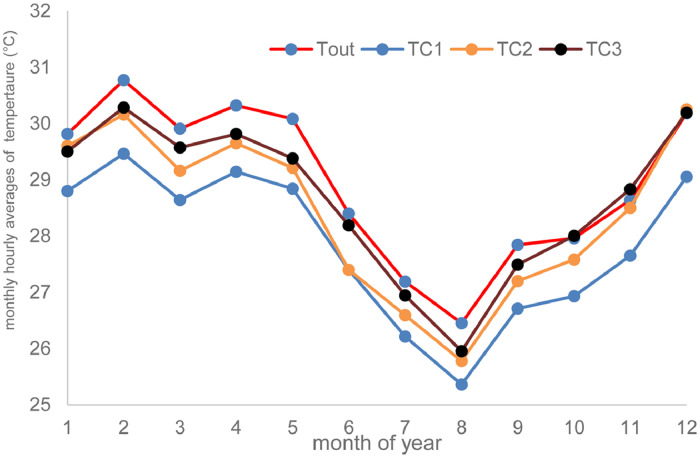
Monthly hourly average of air temperature.

**Data for the month of January**
The changes in the monthly hourly averages are as follows:
29.81 °C ± 3.93 °C for T_out_; 77.85% ± 15.52% for RH_out_
3.05 m/*s* ± 1.64 m/s for V_out_;
28.80 °C ± 1.54 °C for T_C1_; 80.12% ± 5.83% for RH_C1_
29.60 °C ± 1.14 °C for T_C2_; 87.15% ± 2.49% for RH_C2_
29.50 °C ± 1.12 °C for T_C3_; 82.28% ± 3.26% for RH_C3_
**Data for the month of february**
The changes in the monthly hourly averages are as follows:
30.77 °C ± 4.24 °C for T_out_; 75.75% ± 16.00% for RH_out_
3.28 m/*s* ± 1.93 m/s for V_out_;
29.46 °C ± 1.79 °C for T_C1_; 78.66% ± 6.52% for RH_C1_;
30.16 °C ± 1.30 °C for T_C2_; 82.16% ± 3.35% for RH_C2_;
30.28 °C ± 1.29 °C for T_C3_; 78.62% ± 3.68% for RH_C3_;
**Data for the month of march**
The changes in the monthly hourly averages are as follows:
29.91 °C ± 2.72 °C for T_out_; 76.32% ± 8.36% for RH_out_;
3.51 m/*s* ± 2.06 m/s for V_out_;
28.64 °C ± 2.20 °C for T_C1_; 80.32% ± 6.15% for RH_C1_;
29.16 °C ± 1.53 °C for T_C2_; 83.21% ± 2.71% for RH_C2_;
29.57 °C ± 1.58 °C for T_C3_; 79.62% ± 3.06% for RH_C3_;
**Data for the month of april**
The changes in the monthly hourly averages are as follows:
30.32 °C ± 3.28 °C for T_out_; 75.81% ± 10.59% for RH_out_;
3.38 m/*s* ± 2.00 m/s for V_out_;
29.14 °C ± 2.17 °C for T_C1_; 79.77% ± 6.23% for RH_C1_;
29.65 °C ± 1.52 °C for T_C2_; 82.41% ± 2.68% for RH_C2_;
29.81 °C ± 1.60 °C for T_C3_; 79.26% ± 3.10% for RH_C3_;
**Data for the month of may**
The changes in the monthly hourly averages are as follows:
30.08 °C ± 3.00 °C for T_out_; 75.04% ± 9.36% for RH_out_;
3.31 m/*s* ± 1.87 m/s for V_out_;
28.84 °C ± 2.27 °C for T_C1_; 79.41% ± 6.69% pour RH_C1_;
29.21 °C ± 1.45 °C for T_C2_; 82.38% ± 3.00% pour RH_C2_;
29.38 °C ± 1.48 °C for T_C3_; 79.12% ± 3.62% pour RH_C3_;
**Data for the month of june**
The changes in the monthly hourly averages are as follows:
28.40 °C ± 2.49 °C for T_out_; 81.73% ± 8.50% for RH_out_;
3.13 m/*s* ± 1.80 m/s for V_out_;
27.40 °C ± 1.84 °C for T_C1;_ 84.63% ± 5.92% for RH_C1_;
27.84 °C ± 1.31 °C for T_C2_; 86.99% ± 3.00% for RH_C2_;
28.19 °C ± 1.30 °C for T_C3_; 84.53% ± 3.55% for RH_C3_;
**Data for the month of july**
The changes in the monthly hourly averages are as follows:
27.19 °C ± 1.88 °C for T_out_; 85.54% ± 6.95% for RH_out_;
2.97 m/*s* ± 1.65 m/s for V_out_;
26.21 °C ± 1.40 °C for T_C1_; 88.85% ± 4.79% for RH_C1_;
26.59 °C ± 0.94 °C for T_C2_; 93.15% ± 1.40% for RH_C2_;
26.94 °C ± 0.9 °C for T_C3_; 89.79% ± 2.24% for RH_C3_;
**Data for the month of august**
The changes in the monthly hourly averages are as follows:
26.45 °C ± 1.90 °C for T_out_; 87.74% ± 6.03% for RH_out_;
3.057 m/*s* ± 1.99 m/s for V_out_;
25.36 °C ± 1.36 °C for T_C1_; 91.86% ± 3.19% for RH_C1_;
25.78 °C ± 1.06 °C for T_C2;_ 96.34% ± 0.73% for RH_C2_;
25.95 °C ± 1.07 °C for T_C3_; 93.20% ± 1.44% for RH_C3_;
**Data for the month of september**
The changes in the monthly hourly averages are as follows:
27.84 °C ± 2.19 °C for T_out_; 83.27% ± 7.98% pour RH_out_;
3.267 m/*s* ± 1.98 m/s for V_out_;
26.71 °C ± 1.534 °C for T_C1_; 87.98% ± 5.181% pour RH_C1_;
27.20 °C ± 1.13 °C for T_C2_; 94.87% ± 1.492% pour RH_C2_;
27.49 °C ± 1.182 °C for _TC3_; 91.01% ± 3.076% pour RH_C3_;
**Data for the month of october**
The changes in the monthly hourly averages are as follows:
27.96 °C ± 2.51 °C for T_out_; 82.13% ± 8.537% pour RH_out_;
3.088 m/*s* ± 2.128 m/s for V_out_;
26.93 °C ± 1.713 °C for T_C1_; 86.22% ± 5.361% pour RH_C1_;
27.58 °C ± 1.23 °C for T_C2_; 92.38% ± 1.575% pour RH_C2_;
28.00 °C ± 1.234 °C for T_C3_; 87.52% ± 3.124% pour RH_C3_;
**Data for the month of november**
The changes in the monthly hourly averages are as follows:
28.64 °C ± 2.55 °C for T_out_; 80.54% ± 7.967% pour RH_out_;
2.677 m/*s* ± 1.947 m/s for V_out_;
27.65 °C ± 1.889 °C for T_C1_; 84.70% ± 4.945% pour RH_C1_;
28.50 °C ± 1.58 °C for T_C2_; 88.19% ± 2.285% pour RH_C2_;
28.83 °C ± 1.548 °C for T_C3_; 83.73% ± 3.439% pour RH_C3_;
**Data for the month of december**
The changes in the monthly hourly averages are as follows:
30.18 °C ± 2.21 °C for T_out;_ 76.82% ± 8.298% for RH_out_;
2.392 m/*s* ± 2.1 m/s for V_out_;
29.05 °C ± 1.573 °C for T_C1_; 81.05% ± 5.764% for RH_C1_;
30.25 °C ± 1.211 °C for T_C2_; 82.38% ± 3.37% for RH_C2_;
30.19 °C ± 1.271 °C for T_C3_; 79.29% ± 3.852% for RH_C3_;

Monthly hourly averages of the experimental data presented in Table 1 are summarized in Figs. 1 and 2 below.

Fig. 1 shows the evolution of monthly hourly average of air relative humidity. The highest of the monthly-mean air relative humidity is 90,94% in August and the lowest of the monthly-mean minimum air relative humidity is 75,75% in February.

Fig. 2 shows the evolution of monthly hourly average of air temperature. The highest of the monthly-mean air temperature reaches to 30,17 °C in February and the lowest of the monthly-mean hourly air temperatures is 25,36 °C in August.

## 2 Experimental Design, Materials and Methods

### 2.1 Geographic and climatic location of the experimental site

**Fig. 3:**
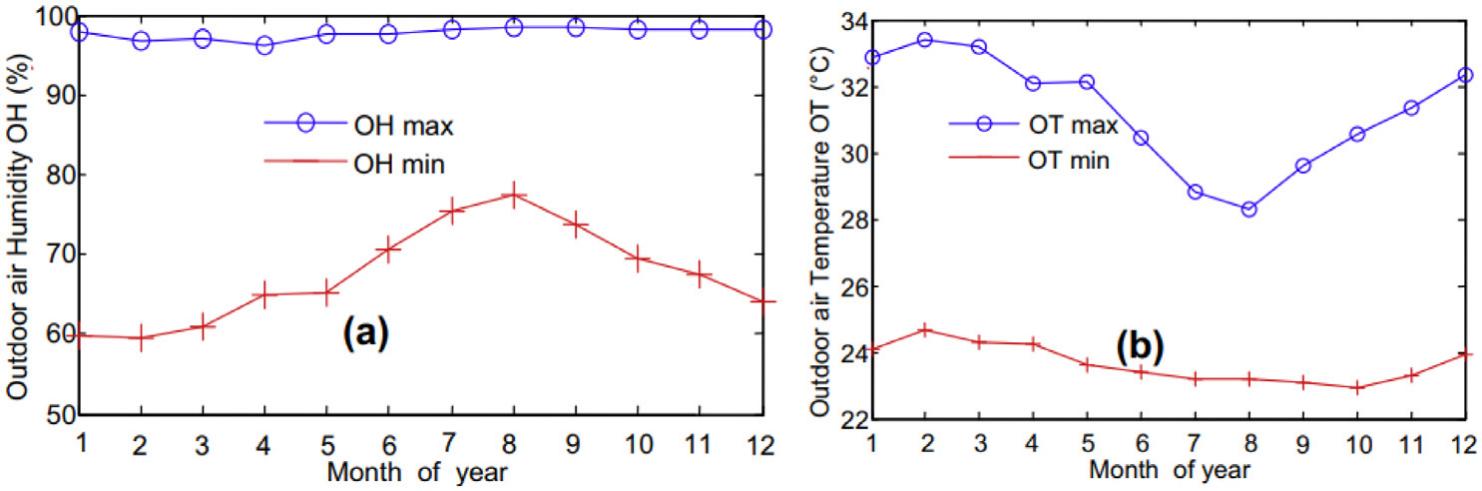
Monthly average of outdoor air relative humidity and temperature [6,7].

The experiment was realised in Douala, a city in Cameroon which is a Central African country. The country extends from the Gulf of Guinea on the Atlantic Ocean to Lake Chad. It extends in length from approximately the 2nd to the 13th degree of North latitude, and in width from the 6th to the 16th degree of East longitude. Its spread and its openness to the sea over 350 km of coastline give it a wide variety of climates [4,5]. The city of Douala is located in a hot and humid zone. Average monthly data are collected over a period of nearly twenty years by the national meteorology of Cameroon [6,7]. Some data are presented on Fig. 3 below.

Fig. 3 shows the evolution of outdoor air relative humidity (a) and temperature (b). In Fig. 3a, the average of maximum relative humidity (OHmax) remains constant throughout the year and is approximately 97%, while the average of minimum relative humidity (OHmin) monthly varies between 59% and 77%. In Fig. 3b, the ambient air temperatures do not vary by an extreme amount. Monthly, the average maximum air temperatures during the day are between 28 and 33 °C and the average of daily minimum air temperature are between 23 and 24 °C. The highest of the monthly-mean maximum air temperatures reaches 33 °C in February and the lowest of the monthly-mean minimum air temperatures is 23 °C in August

### 2.2 Experimental building

**Fig. 4:**
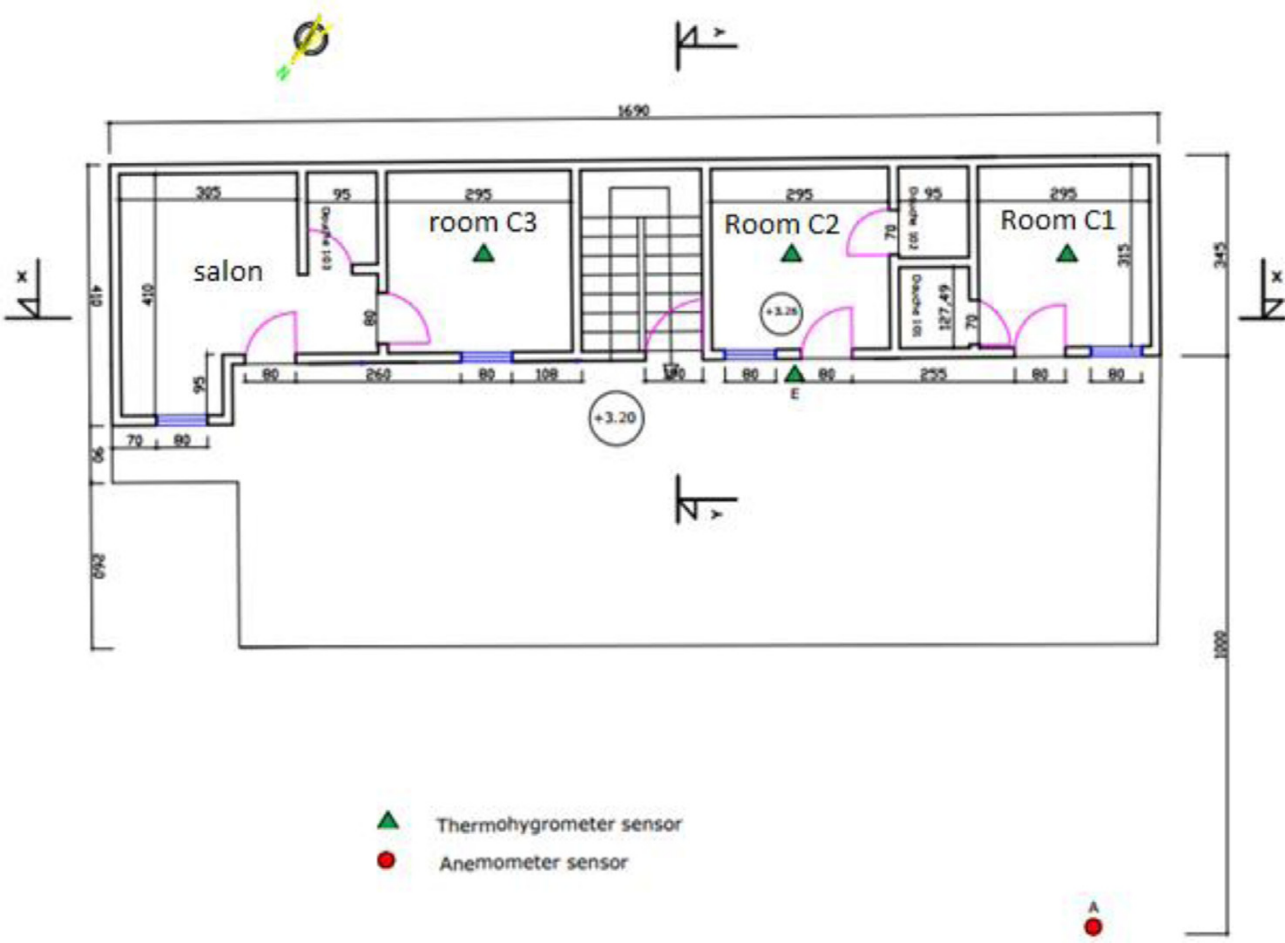
Experimental building plan.

**Fig. 5:**
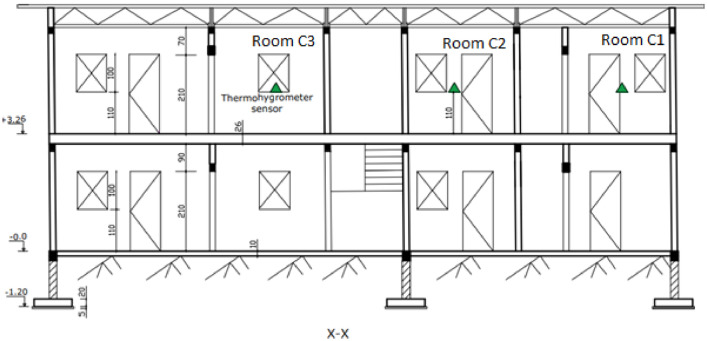
Longitudinal section.

**Fig. 6:**
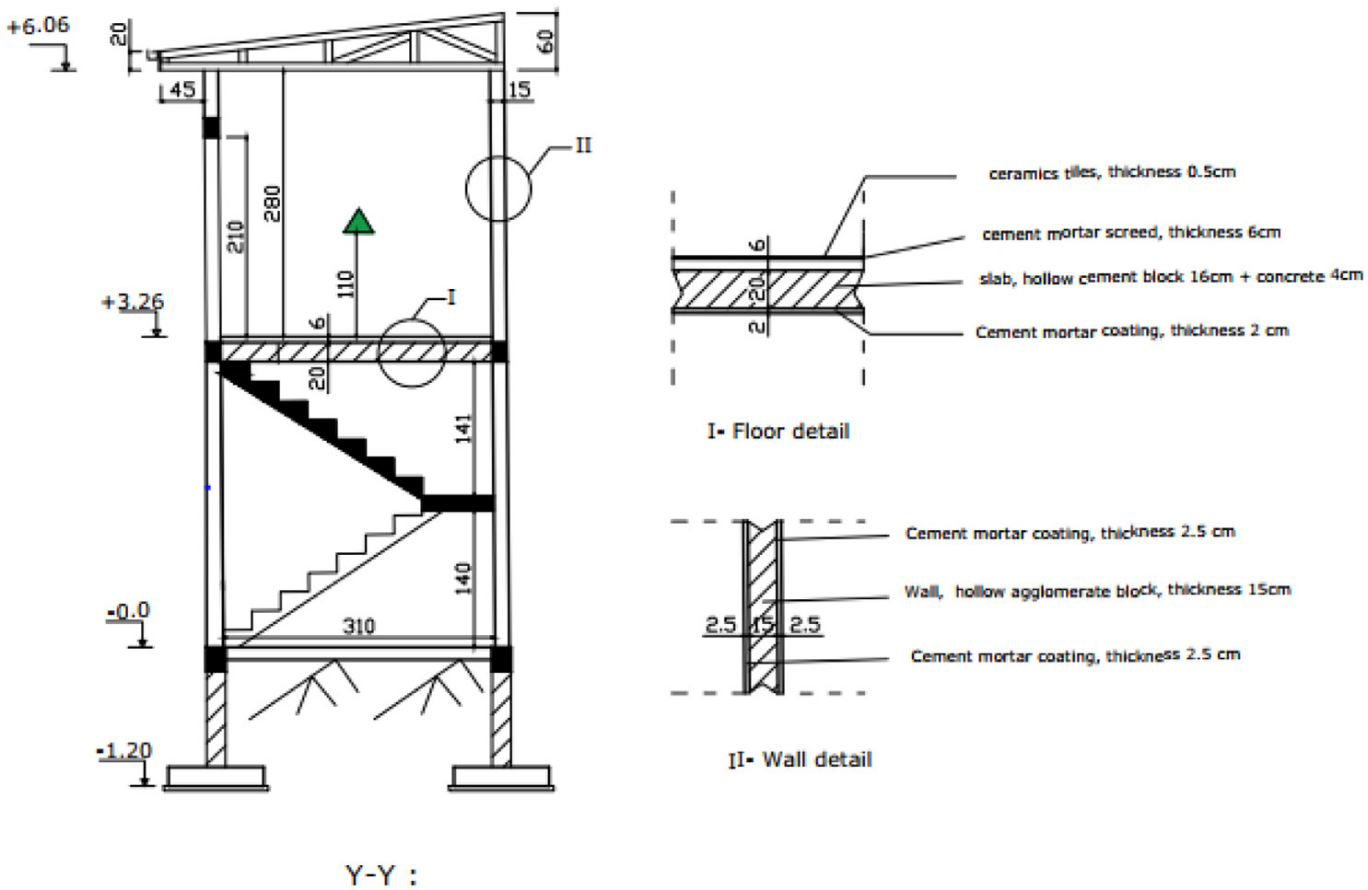
Cross section.

The experimental building is a modern one-storey building with cement block walls. Three almost identical modern rooms were built on the upper floor as shown in Fig. 4, Fig. 5, Fig. 6 attached.

Fig. 4 shows the three (03) experimental rooms which have the same dimensions 2.95 × 3.15 × 3.20 m. The walls are made of 15 × 20 × 40 cm hollow agglomerated concrete blocks. The thickness of the cement mortar plaster is 2.5 cm, the white paint is applied to the interior and exterior walls. The floor is a slab floor covered with a 6 cm thick screed and 0.5 cm thick ceramic tiles. The roof is 0.5 mm aluminium sheets, with a 4 mm thick plywood ceiling. The ceiling height is 2.80 m.

On this plan, the data acquisition equipment is located in the experimentation rooms and in the outside environment:

‐ Room C1 open uninhabited: wooden door and window open.
‐ Closed room C2 uninhabited: wooden door and window closed.
‐ Room C3 inhabited: occupied by one (01) person permanently in the evening with sometimes use of mechanical ventilation, and most often closed and not occupied during the day.

Fig. 5 presents the longitudinal section of the experimental building.

Fig. 6 shows the cross section of elements I and II of the experimental building.

### 2.3 Characteristics of data acquisition equipment

The data acquisition equipment consists of the thermo hygrometers and the anemometer.

#### 2.3.1 Thermo hygrometers

**Photo 1:**
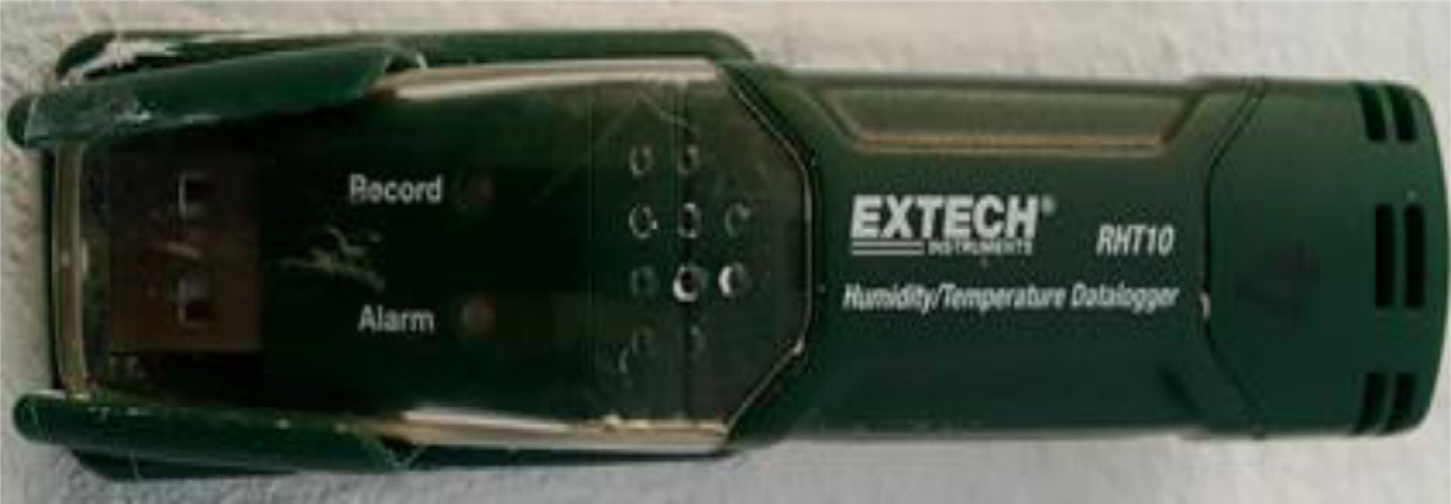
Thermo-hygrometer sensor.

These are relative humidity and temperature data loggers, model EXTECH RHT10 shown in Photo 1 attached.

This logger has the ability to simultaneously measure and save up to 16,000 relative humidity data and 16,000 temperature data.

The characteristics of the sensor are presented as follow:

For Relative humidity,

- General amplitude = 0% to 100%
- Precisions are:
   ± 5.0% for 0% to 20% and 80% to 100%
   ± 3.5% for 20% to 40% and 60% to 80%
   ± 3.0% for 40% to 60%
For temperature

- General amplitude = −40 to 70 °C
- Precisions are:
   ± 2 °C for −40 to −10 °C and + 40 to + 70 °C
   ± 1 °C for −10 to 40 °C

The second class of measuring device is the anemometer.

#### 2.3.2 Anemometer

**Photo 2:**
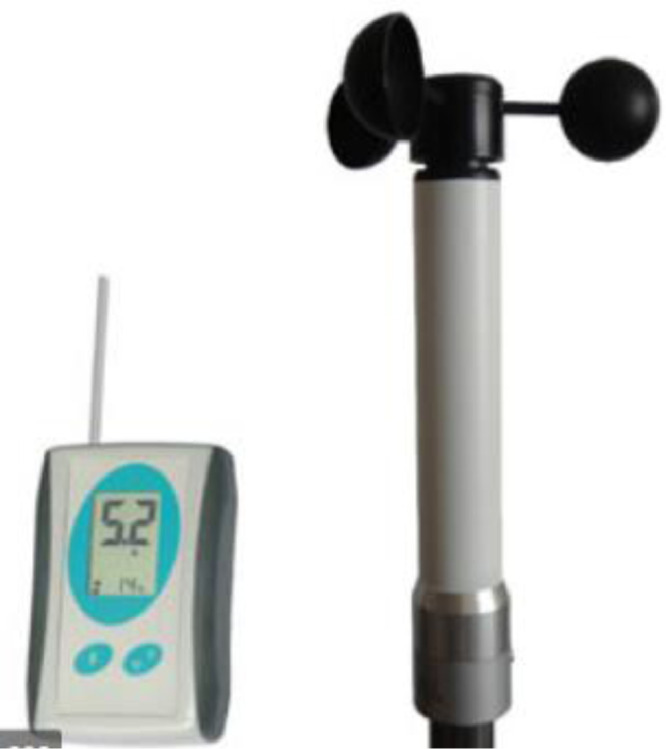
Anemometer.

This is a wind speed sensor. In this work, the "WINDVISU" model shown in Photo 2 below is used.

The characteristics of the anemometer are presented as follow:

- General amplitude = 0 to 50 m/s
- Precision is ± 3%
- Resolution is 0.1

#### 2.3.3 Computer

A laptop computer was used for data storage and data processing.

#### 2.3.4 The inverter

An inverter is used to ensure the operation of the anemometer in case of short power failure.

### 2.4 Experimental protocol

Data were collected using thermohygrometers, then stored in the computer.

#### 2.4.1 Data acquisition with thermohygrometers

Inside each of the three rooms (C1, C2 and C3), thermohygrometers are hung from the ceiling, 1.10 m from the floor, in compliance with the ASHRAE 55 standard [1] on instruments for measuring thermal comfort parameters in buildings. In the outdoor environment, there is another thermohygrometer responsible for acquiring the temperature and related humidity of the outdoor air. The data acquisition step is set at 1 h.

#### 2.4.2 Data acquisition using the anemometer

The anemometer is placed above the roof of the building. It is set at a recording step of 1 h. A memory card is inserted. The card can record data for one year.

#### 2.4.3 Data storage

**Photo 3:**
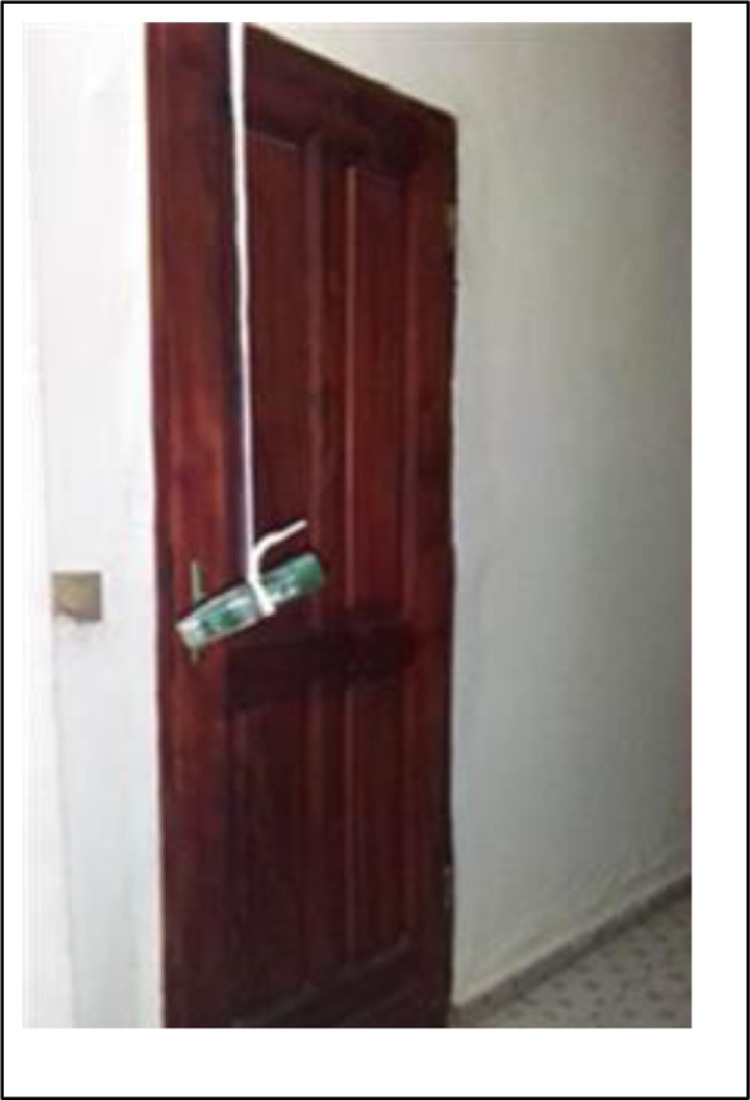
Thermo-hygrometer sensor 1.10 m from the floor.

**Photo 4:**
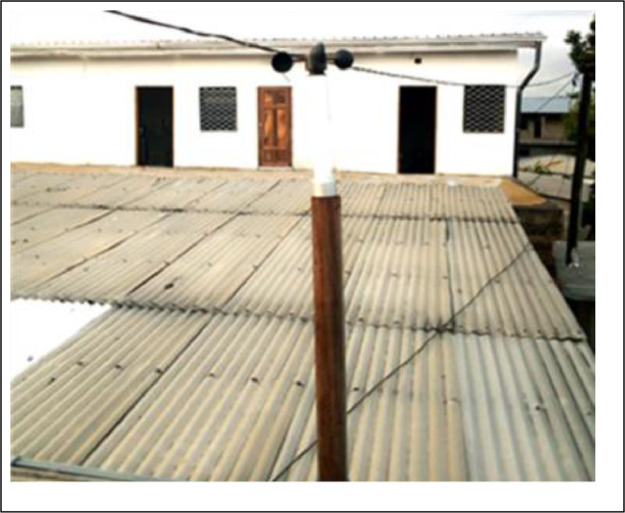
Anemometer sensor 10 m from the experimental building.

Time data is collected for a period of one year, from January 2019 to December 2019. Data is immediately downloaded and stored on a dedicated computer Photos 3 and 4.

## Ethics Statement

All respondents participating in the experiment process provided their informed consent before participating.

## CRediT authorship contribution statement

**Thomas Janvier Matongo:** Project administration, Conceptualization, Investigation, Writing – original draft. **Jean Gaston Tamba:** Supervision, Formal analysis, Validation, Writing – review & editing. **Léopold Mba:** Methodology, Data curation, Visualization, Writing – review & editing. **Emmanuel Yamb:** Supervision, Formal analysis, Validation, Writing – review & editing.

## Declaration of Competing Interest

The authors declare that they have no known competing interests.

## Appendix Supplementary materials

Image, application 1
